# Pediatric Post-Pump Chorea: Case Report and Implications for Differential Diagnosis

**DOI:** 10.3390/children11091060

**Published:** 2024-08-29

**Authors:** Elisa Rossi, Concetta Strano, Ilaria Cortesia, Francesca Torta, Mirella Davitto Bava, Irene Tardivo, Marco Spada

**Affiliations:** 1 Department of Pediatrics, University of Turin, 10126 Turin, Italy; elisa.rossi@unito.it (E.R.); concetta.strano@unito.it (C.S.); ilaria.cortesia@unito.it (I.C.); mdavittobava@cittadellasalute.to.it (M.D.B.); marco.spada@unito.it (M.S.); 2 Department of Pediatrics, Regina Margherita Pediatric Hospital, 10126 Turin, Italy; ftorta@cittadellasalute.to.it

**Keywords:** post-pump choreoathetosis, pediatric chorea, cardiac surgery, neurological complications

## Abstract

Background: Chorea is a neurological disorder characterized by random, fluid movements that may affect the limbs, trunk, neck, or face. In children, Sydenham’s chorea (SC) is the most common cause of acute chorea, mainly following group A beta-hemolytic streptococcal (GABHS) infection. Other autoimmune and metabolic disorders may also cause chorea. Case presentation: We report the case of a 6-year-old girl who developed chorea following cardiac surgery for mitral insufficiency. One week after discharge, the patient presented with right-sided hyposthenia, slower speech, mild dysarthria, and sialorrhea. Brain MRI and intracranial MRI angiography revealed a small vascular lesion consistent with a microembolic event. Extensive diagnostic investigations, including serum panels for autoimmune encephalitis, neurotropic viruses, and metabolic disorders, were negative. Conclusions: Considering the patient’s history, clinical course, and the exclusion of other potential causes, a diagnosis of post-pump chorea was made. This case underlines the importance of a thorough differential diagnosis in pediatric chorea and highlights post-pump chorea as a significant postoperative complication in pediatric cardiac surgery. The patient’s motor symptoms improved with symptomatic treatment, and follow-up showed good recovery without neurological sequelae.

## 1. Introduction

Chorea is a neurological condition characterized by random and flowing movements with a dance-like appearance, usually switching quickly and irregularly between the limbs, trunk, neck, or face [1,2]. An insidious onset in children younger than 5 years old is more linked to genetic causes. Conversely, symptoms usually begin abruptly in acquired forms after 5 years of age.

In the pediatric population, Sydenham’s chorea (SC) represents the most common cause of acute chorea. Its etiology is due to an autoimmune sequelae that occurs after a group A beta hemolytic streptococcal (GABHS) infection. The age of onset is between 5 and 15 years, mostly in females [3,4,5,6]. SC has mainly a bilateral involvement, but it can be unilateral in about 20–35% of cases [4,7]. Other neurological, and also psychiatric, findings can be associated with chorea movements, such as hypotonia, psychomotor agitation, hypometric saccades, anxiety, obsessive compulsive symptoms, and attention and hyperactivity syndrome [8]. Throat cultures showing streptococcal infection, positive anti-streptolysin-0, or anti-DNAse antibodies lead to diagnosis [9,10]. Clinical resolution occurs in 1–6 months even if the recurrence of symptoms in 20–60% of cases is seen within 2 years from onset [11,12].

Autoimmune disorders, such as autoimmune encephalitis and systemic lupus erythematosus syndrome (associated with antiphospholipid syndrome), seem to have a large impact on the onset of chorea in children, and females are often the most affected [13,14]. Around 1–3% of patients with SLE and APS develop chorea. It is usually generalized, and psychosis and behavioral disturbances are quite featured, with a resolution course predominant [3].

As well as autoimmune cases, viral encephalitis also plays a role in the genesis of this neurological abnormality. Herpes simplex virus, mumps, varicella parvovirus B19, and measles are typically responsible for neuronal involvement and the possible subsequent onset of chorea. In addition, the presence of other agents such as Borrelia and Toxoplasma should be excluded because of their neurotropism [15].

Drug toxicity should be considered in the diagnostic approach of choreic disorders. A careful medical history is required for the potential intake of dopamine-receptor-blocking agents or antiparkinsonian or antiepileptic drugs.

Several inborn metabolic diseases may also present with chorea, notably some specific organic acid disorders, such as glutaric acidemia type 1 and methylmalonic acidemia [9].

When acute hemichorea is reported, it is important to evaluate common complications of type 1 and 2 diabetes. Another peculiar etiology of unilateral chorea, though less frequent in children, is vascular stroke. Most cardioembolic strokes in pediatric patients occur in the setting of cardiac surgery, as survival after this procedure has increased [16].

For this reason, in the last few years, more attention has been paid to “post-pump chorea” (PPC), a complication of pediatric open-heart surgery (particularly when cardiopulmonary bypass is used) [17]. Currently, it is still not clear whether pathogenesis is due to microemboli or to an inadequate restoration of cerebral circulation after operation. PPC occurs within 2 weeks after surgery [18]. Signs and symptoms often improve or fade in a few weeks or months [19].

In this report, we describe a case of chorea in a 6-year-old child who underwent heart surgery for moderate–severe mitral insufficiency, with the aim of providing greater awareness about the differential diagnosis among the many causes of chorea in the pediatric population, giving particular importance to risk factors and the clinical presentation of patients who undergo our procedure.

## 2. Case Report

A 6-year-old girl, born at term by C-section, Small for Gestational Age (SGA), and with a birth weight of 2450 g, has a medical history of moderate–severe mitral insufficiency due to a prolapse of the anterior mitral leaflet and severe hypoplasia of the posterior one. At the age of 6 years old, she underwent cardiac surgery for valve repair in extracorporeal circulation (total ECC time of 6 h and cooling temperature of 32 °C).

About two weeks after the surgery, the patient was taken to the Emergency Room of “Regina Margherita” Pediatric Hospital of Turin for hyposthenia of the right upper limb and the right lower one associated with slower speech, mild dysarthria, and sialorrhea.

Neurological evaluation revealed repeated, involuntary, non-finalistic, choreo-athetotic-like distal movements involving the right hand and foot that sporadically became wider until they involved the ipsilateral arm and leg too. Light hyposthenia of the right upper and lower limb and a right-hand grasp deficit were reported too. There were no abnormalities in sensory function, deep tendon reflexes, and cranial nerve function.

**Figure 1:**
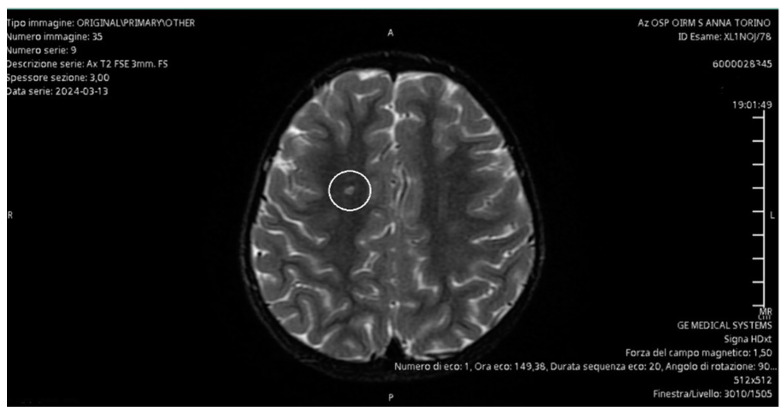
Magnetic resonance imaging scan shows a small vascular lesion on a probable microembolic basis at the right semioval center.

In the regime of emergency, an MRI of the brain and an intracranial MRI angiography were performed, with evidence of a small vascular lesion on a probable microembolic basis at the right semioval center. This allowed us to exclude the presence of brain masses and stroke as causes of chorea, as the clinical manifestations were ipsilateral to the microembolic lesion (Figure 1).

An electroencephalogram (EEG) was also performed, revealing initial signs of bilateral parieto-occipital slow dysrhythmia. This was monitored with subsequent serial electroencephalograms and gradually normalized.

Further investigations allowed us to exclude the main causes of chorea in children. Above all, the hypothesis of rheumatic chorea was excluded due to negative tests related to a previous group A beta-hemolytic streptococcus infection.

Rachicentesis was not performed due to ongoing warfarin therapy, but serum panels for dysimmune encephalitis and serum virological tests within normal limits allowed us to exclude autoimmune and infectious chorea too.

Moreover, her medical history had not revealed the prescription of any pharmacological agents potentially interfering with movement, and drug-induced chorea was finally ruled out by an extensive toxicological work-up in plasma and in urine, which did not show any abnormal finding.

To investigate inherited metabolic conditions that may present with chorea, specific analyses were then performed, including the determination of leucine concentration in plasma (127 umol/L; normal values < 250 umol/L) by amino acid analysis, the determination of total plasma homocysteine (12 umol/L; normal values < 15 umol/L), the determination of serum ceruloplasmin (31 mg/dL; normal values: 20–40 mg/dL), the determination of plasma lactate (1.2 mmol/L; normal values < 2.5 mmol/L), the determination of serum uric acid (3.8 mg/dL; normal values < 6 mg/dL), and the determination of urine organic acids, which did not reveal any abnormal excretion of methylmalonic acid, propionic acid, and glutaric acid. Finally, the analysis of the acylcarnitine profile by tandem mass spectrometry and the measurement of biotinidase enzyme activity in dried blood spots were also found to be normal.

Refer to Table 1 for detailed information on the analyses performed.

Medical treatment with warfarin, prednisone, and oral amoxicillin was administered until the rheumatic etiology of chorea was excluded, and antibiotic therapy was therefore suspended. The patient was also offered neuro-rehabilitation including physiotherapy and speech therapy assessment.

In consideration of anamnestic data and the results of the tests, a diagnostic hypothesis of post-pump chorea was established.

As a result, there was a remarkable improvement in motor symptoms and radiological data, remotely controlled, with the expected evolution of the microembolic alteration, which is commonly found in patients undergoing extracorporeal circulation.

The patient made a good overall recovery in two weeks, after which the treatment was gradually withdrawn, and she was discharged without neurological consequences.

## 3. Discussion

We report the case of a 6-year-old girl admitted to the Pediatric Emergency Department in Turin in March 2024 with neurological symptoms characterized by choreoathetotic movements. These symptoms manifested approximately two weeks after cardiac surgery for mitral valve repair. Several diagnostic hypotheses were considered and subsequently excluded before the diagnosis of post-pump chorea was established. Symptomatic treatment was initiated, resulting in a resolution of the clinical condition without any neurological sequelae.

The diagnostic pathway for our patient involved evaluating several hypotheses. The brain MRI and intracranial MRI angiography allowed us to exclude brain masses and ischemic lesions responsible for the symptoms. The instrumental examination showed evidence of a microhemorrhagic lesion located ipsilateral to the body side affected by the neurological disturbance. After consulting with the radiologists who performed the investigation, it was determined that the microhemorrhagic lesions may be consistent with the result of extracorporeal circulation used during cardiac surgery. However, it is also important to consider the increased risk of embolism associated with the presence of a prosthetic device at the site of valve repair.

Given the age of our patient (older than five years) and the symptoms at presentation, we focused on acquired chorea. From an epidemiological point of view, the first hypothesis we considered was Sydenham’s chorea. Considering the negative result of the laboratory tests and the throat swab for group A beta-hemolytic streptococcus infection, this hypothesis was excluded. Although the patient presented with mitral insufficiency, which appeared to be compatible with rheumatic fever, the valvular defect had been first diagnosed more than three years earlier. In addition, according to the cardiologists, the morphology of the valve did not appear to be consistent with rheumatic fever. Finally, the child presented with unilateral neurological symptoms at onset, whereas Sydenham’s chorea more commonly involves both sides.

Subsequently, to exclude both autoimmune and infectious etiologies, the serum panel for dysimmune encephalitis and the serology panel for neurotropic viruses, including Borrelia, were performed and found to be negative.

Chorea can also characterize the clinical presentation in a limited percentage of children suffering from certain inborn metabolic diseases, including aminoacidopathies such as homocystinuria and maple syrup urine disease; organic acidemias such as glutaric acidemia type 1, biotinidase deficiency, methylmalonic acidemia, and propionic acidemia; purine metabolism disorders such as Lesch–Nyhan disease; copper disorders such as Wilson disease; and pyruvate metabolism disorders such as pyruvate dehydrogenase deficiency. To investigate these conditions, we performed a specific biochemical work-up based on the analysis of amino acids, homocysteine, acylcarnitines, organic acids, biotinidase activity, uric acid, ceruloplasmin, and lactate. This metabolic screening allows for the identification of the majority of inborn metabolic disorders presenting with chorea [20].

After performing all the diagnostic investigations and considering the patient’s medical history and clinical course, the diagnosis of post-pump chorea was reached by exclusion (Table 1). According to the cases reported in the literature, the symptoms presented by the patient are indicative of typical chorea. The onset of symptoms within two weeks after surgery, the presence of a symptom-free interval, and the bilateral characteristic of the choreoathetotic movements are consistent with findings documented in the literature of typical PPC.

Post-pump chorea is one of the most concerning postoperative neurological complications for pediatric patients undergoing cardiac surgery. Although post-pump chorea was first described by Bjork and Hultquist in the 1960s, the underlying pathophysiological mechanisms remain largely unknown. The most widely accepted hypothesis suggests that neurological complications could be due to hypoxic–ischemic events or disturbances in the cerebral blood flow following reperfusion after prolonged hypothermia in patients undergoing extracorporeal circulation [21].

Early studies reported an incidence rate of up to 18% [22], but, according to more recent research, this has fallen to between 0.6 and 1.7% [23].

Choreoathetotic movements typically occur within two weeks of the surgical procedure, as in the case of our patient, whose mother noticed hyposthenia in her right arm about ten days after surgery [18].

Our patient’s symptomatology initially involved the right hemilatum, with right-hand hypotilization and dragging of the right lower limb, associated with slowed speech. Gherpelli et al. in 1998 observed 647 children who had undergone cardiac surgery and described how choreic movements predominantly affected the limbs bilaterally as well as the orofacial musculature [19].

Over the years, numerous risk factors associated with the development of neurological complications have been identified, including the duration and degree of hypothermia during the surgical procedure, the induction of intraoperative cardiac arrest with extracorporeal circulation, the patient’s age (with increased vulnerability under one year of age up to 5–6 years) and weight, and the presence of cyanotic heart disease [17,23,24].

Our patient is a 6-year-old girl with poor ponderal-staturo growth and a diagnosis of severe mitral insufficiency approximately one year prior. The surgical procedure was carried out in two stages, with a double induction of extracorporeal circulation under moderate hypothermia (32 °C).

As for diagnostic investigations, instrumental examinations are often normal in children with post-pump chorea. Electroencephalographic findings are negative in most cases, although some patients exhibit a diffuse slowing of the electrical activity, as observed in our patient [19,21].

Additionally, children with an onset of neurological complications after cardiac surgery have been evaluated using CT and diffusion- and perfusion-weighted MRI of the brain, with findings that can be highly variable. During the acute phase, MRI may often be normal or show a pattern of multiple microembolic infarcts, anomalies in the basal ganglia, or diffuse atrophy [19,25,26]. Consistent with the literature, our patient was studied using contrast-enhanced MRI and intracranial MRI angiography, which revealed the presence of a vascular lesion in the right centrum semiovale and punctiform microhemorrhagic foci as a consequence of extracorporeal circulation.

The available treatment of post-pump chorea is symptomatic; since the 1960s, multiple attempts have been made with tetrabenazine, valproate, levetiracetam, and carbamazepine without any benefit. Currently, the only therapy routinely used in clinical practice is neuroleptics, such as Haloperidol, without any evidence; however, this could modify the evolution of the disease [18].

Typically, the symptoms resolve within 2–4 weeks, although cases have been described with long-term neurological sequelae, ranging from memory, language, and motor function deficits to more severe clinical presentations such as long-term motor and cognitive disabilities [18,21]. A pediatric neuropsychiatric follow-up was established for our patient, including electroencephalographic investigations and the monitoring of academic learning, to analyze the long-term consequences.

## 4. Conclusions

This case highlights the significance of a thorough differential diagnosis in pediatric chorea, emphasizing the importance of considering even less common diagnoses based on a patient’s medical history and clinical presentation. The diagnosis of post-pump chorea was established by exclusion, following a comprehensive evaluation of several diagnostic hypotheses.

Neurological complications following cardiac surgery remain a significant cause of morbidity and mortality in pediatric patients. The child of our case report presented several risk factors, despite the hypothermia not being severe. Further studies are needed, particularly regarding the management and treatment of this complication, to promptly recognize the clinical presentation and address it adequately. Additionally, the use of advanced imaging techniques and neurophysiological monitoring may provide further insights into the pathophysiological mechanisms underlying these complications.

Finally, multidisciplinary collaboration between surgeons, pediatric neuropsychiatrists, radiologists, and pediatricians is essential for the early recognition of choreic symptoms and identification of the underlying etiology. This approach may allow early treatment, helping to minimize long-term complications.

## Figures and Tables

**Table 1:** Main differential diagnosis of chorea and the corresponding diagnostic biomarkers performed.

Differential Diagnosis of Chorea			
Disease	Diagnostic Biomarkers	Disease	Diagnostic Biomarkers
Rheumatic-Dysimmune Chorea		Metabolic Chorea	
Rheumatic Fever	Anti-DNAse antibodies, TASLO, pharyngeal swab for group A beta-hemolytic streptococcus infection	Aminoacidopathies (such as homocystinuria and maple syrup urine disease)	Amino acids and total homocysteine in plasma
		Organic acidemias (such as glutaric acidemia type 1, biotinidase deficiency, methylmalonic acidemia and propionic acidemia)	Organic acids in urine, acylcarnitines and biotinidase activity in dried blood spots
Autoimmune Systemic Diseases	Antiphospholipids, Lupus Anticoagulant (LAC), ANA, anti-beta2 glycoprotein antibodies		
		Purine metabolism disorders (such as Lesch–Nyhan disease)	Uric acid in plasma
Dysimmune Encephalitis	Anti-neuronal antibodies (NMDA, MOG), anti-AMPAr, anti-GABAr B1, anti-LG1, anti-CASPR antibodies		
		Wilson disease	Ceruloplasmin in plasma
		Pyruvate metabolism disorders (such as pyruvate dehydrogenase deficiency)	Lactate in plasma
Infectious Chorea		Structural Basal Ganglia Chorea	
Infectious Diseases	Serology for main neurotropic viruses: Lyme disease, EBV, Parvovirus, Measles, Chickenpox, Toxoplasma + Borrelia, Mycoplasma, HIV.	Brain masses Vascular diseases Moya Moya Trauma	Brain MRI Intracranial MRI-angiography
Toxic Chorea		Miscellaneous	
Heavy Metal and Drug Intoxication	Serum level of heavy metals (carbon monoxide, manganese)	Nutritional deficit	Sodium, potassium, calcium
		Vitaminic deficiency	B12 serum level
		Thyroid disease	TSH, fT4, fT3
Anamnestic Chorea			
Drug-Induced		Post Pump	
Clinical History			

## Data Availability

The original contributions presented in the study are included in the article, further inquiries can be directed to the corresponding author/s.
